# Supplementary material for: Are carbon emissions trading and green financial instruments synergistic? -Comprehensive quantitative research based on content analysis
Source: PLoS One. 2024 Mar 7;19(3):e0298601. doi: 10.1371/journal.pone.0298601 (PMC10919601; doi:10.1371/journal.pone.0298601)
Supplement: S1 Data — (ZIP) [file pone.0298601.s001.zip › Data/地级市Z_F工作报告.zip!/╔╜╢1⁄2/╫═▓⌐╩╨/└┤╫╘╣1⁄2╓┌║┼í╛┬φ┐╦╩2╛▌═°í┐_2017.docx]

各位代表：

现在，我代表市人民政府，向大会作政府工作报告，请予审议，并请各位政协委员和列席会议的同志提出意见。

一、过去五年政府主要工作

过去的五年，在中共淄博市委的坚强领导下，全市上下深入贯彻党的十八大和十八届三中、四中、五中、六中全会以及习近平总书记系列重要讲话重要批示精神，围绕“一个定位、三个着力”，主动适应经济发展新常态，积极应对经济下行带来的压力与挑战，团结进取，奋力攻坚，顺利完成“十二五”规划及市十四届人大一次会议确定的各项目标任务，谱写了老工业城市转型发展新篇章。

——五年来，我们始终坚持科学发展不动摇，综合实力跨上新台阶。2016年，全市地区生产总值实现4412亿元，是2011年的1.5倍；一般公共预算收入达到345.4亿元，是2011年的1.7倍；城镇和农村居民人均可支配收入分别达到36436元和15674元，是2011年的1.56倍和1.67倍。我市先后蝉联全国文明城市、国家卫生城市、全国“双拥模范城”和全国社会治安综合治理优秀市，成功创建为国家森林城市、知识产权示范城市和国土资源节约集约模范市，并被确定为全国生态文明先行示范区、国家扶贫改革试验区、全国战略性新兴产业区域集聚发展试点和国家新型城镇化综合试点，淄博高新区列入山东半岛国家自主创新示范区。

——五年来，我们牢牢把握结构调整这条主线，老工业城市转型升级取得重要进展。凝聚建设工业强市强大合力，研究出台“工业强市30条”“创新发展30条”等政策措施和工业精准转调“1+N”实施方案，在强化创新驱动、建设重大项目、培育骨干企业、推进“铸链工程”等关键环节持续用力，累计完成技术改造投资5360亿元，六大高耗能行业占规上工业增加值比重累计下降3个百分点，高新技术产业占比提高6.5个百分点，以新能源汽车、生物医药、电子信息、节能环保等产业为代表的新动能加速形成。实施淄博经济开发区、齐鲁化工区（一区四园）、主城区南部区域等重大生产力布局，加快推进省级经济开发区“二次创业”，组织实施产业空间布局调整，产业集约集群发展态势更加清晰。现代物流、金融保险、创意设计、电子商务、信息咨询等现代服务业加快发展。粮食生产实现“十四连丰”，农业综合效益和竞争力逐年提高。全市三次产业占比由2011年的3.6∶60.2∶36.2调整为3.4∶52.5∶44.1。

——五年来，我们强力推进环境保护和生态建设，区域生态环境质量显著改善。以铁的决心和措施突破生态环境瓶颈，集中打响空气异味综合整治和孝妇河流域综合治理两场攻坚战，全力推进重点区域、重点行业深度治理，累计完成环境治理工程7035项;创新实施总投资107亿元的绿动力提升工程，实施工程项目2394个，年可削减燃煤消耗1000万吨。2016年，全市空气质量优良天数和“蓝天白云、繁星闪烁”天数分别达到183天和225天，比启动实施新标准的2014年增加63天和90天；SO2、NO2、PM10和PM2.5平均浓度比2014年分别改善50.4%、16.9%、18.3%和22.9%。着力构建“清水润城”生态水系，累计治理骨干河道360公里，新建改造城镇污水处理厂21座，主要河流断面COD和氨氮平均浓度比2011年分别改善24%和43.3%。大力推进主城区东南部等重点区域生态修复，高标准建设马踏湖、五阳湖、齐盛湖公园、孝妇河湿地公园等一批生态景观，全市森林覆盖率和建成区绿化覆盖率分别达到37%和45.2%。博山区、沂源县列入国家重点生态功能区。

——五年来，我们扎实推进统筹融合发展，新型城镇化迈出坚实步伐。确立了建设“现代化组群式大城市”发展定位和“四位一体、组群统筹、全域融合”城市工作思路，常住人口城镇化水平达到69.1%，比2011年提高5.1个百分点。五年新建改造干线公路637公里、城市道路393公里。淄博新区路网和水系建成贯通，商务金融中心主体基本建成，市规划展览馆、图书馆新馆、柳泉中学等公共服务项目竣工投用，文化中心、教育医疗及高端服务业领域的一批重大项目加速推进；启动高铁新城建设，济青高铁、济青高速、滨莱高速、S102淄博段以及马南路等一批新建改建骨干道路集中开工；策划实施“三改一建”工程，棚户区改造和老旧住宅小区整治受益群众达9.4万户。2016年，127项市及全市统筹城建重点项目完成投资224亿元。扎实开展城乡环境综合整治，深入推进美丽乡村建设，建成236个功能完善、舒适宜居的新型农村社区，农村公路通车里程突破1万公里，解决81.2万农村居民饮水安全问题，改造农村危房8879户，完成26.7万户农村旱厕改造，城乡环卫一体化实现全覆盖。

——五年来，我们坚定不移深化改革扩大开放，区域发展活力持续增强。承接国家和省级改革试点126项，推广自主创新重大改革经验30项。加大简政放权力度，着力建设“三最”城市，在全省动态保持市级行政审批事项最少、时限最短、收费最低，我市创新实施联动审批提高行政服务效率的做法被国务院办公厅通报表扬。深化商事制度改革，五年新登记市场主体22万户。扎实推进供给侧结构性改革，“去产能”任务如期完成，存量商品房去化周期保持在合理区间，企业“去杠杆”和“降成本”政策全面落实。深化投融资体制改革，设立9支市级政府引导基金，农村信用社银行化改革顺利完成，全市在齐鲁股交中心挂牌企业达到416家，新增上市企业5家、新三板挂牌企业44家。行政管理体制、农村产权制度、财税体制、事业单位、基础教育、医疗卫生等重点领域改革扎实推进。努力扩大更高水平对外开放，五年实际到位外来投资2159.8亿元，累计实现外贸进出口2707亿元，汉能、国金、中船重工、远成、传化等一批重大招商项目落地开工。全市省级以上研发机构达到297家、博士后科研工作站和创业示范平台56家、院士工作站75家，数量均居全省前列。淄川、博山、周村、沂源等区县重大区域政策争取和地炼企业争取进口原油指标顺利实现。陶博会、新材料技术论坛等品牌展会影响力不断提升。

——五年来，我们积极顺应人民群众新期待，民生和社会建设全面加强。以决战决胜力度推进脱贫攻坚，精准识别认定的市定标准贫困人口基本实现“两不愁、三保障”。城镇登记失业率控制在3%以内，覆盖城乡的社会保障体系基本建立。加力推进文化名城建设，齐文化传承创新示范区建设列入省“十三五”发展规划，齐国故都列入全省十大文化旅游目的地品牌，齐文化研究院挂牌成立，文化旅游融合发展“十大工程”有序实施，文化产业增加值占比达到4.17%。坚持教育优先发展战略，新建改建学校219所，中小学校舍安全工程、解决“大班额”“全面改薄”等重点工作走在全省前列，所有区县全部创建为国家义务教育均衡县（区）。市中心医院西院区、市妇幼保健院新院区等重大项目加快实施。城乡卫生计生服务体系日益完善，生育水平保持稳定。食品药品安全水平稳步提升。参加奥运会等重大赛事取得优异成绩。国防动员和民兵预备役工作扎实推进。构建完善社会治理“三四二”工作格局，社会大局持续和谐稳定。统计、物价、住房公积金、广播电视、民族宗教、外事侨务、对台、防震减灾、气象、人防、仲裁、史志、档案、关心下一代、妇女儿童、老龄、慈善、残疾人、红十字、对口支援等各项事业都取得了新成绩、实现了新发展。

——五年来，我们始终高度重视政府自身建设，法治政府和服务型政府建设取得新成效。深入开展党的群众路线教育实践活动、“三严三实”专题教育和“两学一做”学习教育，自觉用习近平总书记系列重要讲话精神武装头脑、指导实践。创新“市长市民面对面”等制度安排，建成运行全市统一的民生热线服务平台。组建市政府重大行政决策专家咨询委员会和法律顾问委员会，开通市行政执法网，编制公布市及区县行政权力清单和责任清单，建成市级公共资源交易平台。自觉接受市人大及其常委会的法律监督和政协的民主监督，虚心听取各民主党派、工商联和社会各界意见，认真办理人大代表建议和政协委员提案。全面贯彻中央八项规定和省市委加强党风廉政建设的一系列部署要求，切实改进作风、狠抓落实，政府公信力、执行力不断提升。

刚刚过去的2016年，我们按照市委“一个定位、三个着力”总体思路和“十个新突破”工作重点，积极应对经济下行，全力推进转型升级，在产业结构调整和发展动能转换方面取得了积极进展。全市地区生产总值和一般公共预算收入分别比上年增长7.7%和9.2%，固定资产投资、社会消费品零售总额和外贸进出口总额分别增长13.4%、10.5%和10.5%，城镇居民和农村居民人均可支配收入分别增长7.8%和7.9%，经济社会保持稳中向好发展态势，实现了“十三五”良好开局。

各位代表！过去的五年，在中共淄博市委的坚强领导下，全市上下团结一心，谱写了在困境中奋进、在转型中发展的壮丽篇章。这五年，是老工业城市加速转型发展的五年，是淄博新的竞争优势不断凝聚的五年，是人民群众得到更多实惠的五年，是我们在全面建成小康社会道路上奋力前进的五年。五年的成就，得益于党中央、国务院和省委、省政府的科学决策和亲切关怀，得益于市委的正确领导，得益于全市各级人大、政协与社会各界的有力监督和大力支持，得益于广大干部群众负重奋斗、不懈努力。在此，我代表市人民政府，向全市广大干部群众，向各民主党派、工商联、各人民团体和社会各界人士，向中央、省驻淄单位、驻淄部队和武警官兵、政法干警，向海内外所有关心支持淄博改革发展的同志们、朋友们，表示衷心感谢并致以诚挚敬意！

在总结成绩的同时，我们也清醒认识到，我市经济社会发展还面临一系列困难和问题，主要是：经济结构性矛盾依然突出，提升产业层次、强化创新驱动、加快动能转换任务艰巨；主要污染物排放总量仍然较大，资源环境约束加剧，生态环境治理任重道远；公共服务供求矛盾较大，社会和民生领域存在不少“短板”；社会组织结构和利益诉求更加多元，安全生产、金融运行等领域防风险压力加大，社会治理面临许多新课题新挑战；各项改革亟待进一步深化，政府自身建设仍需切实加强，等等。对这些问题，我们一定高度重视，采取更加扎实有力的措施，积极认真解决。

二、今后五年工作的指导思想和主要任务

今后五年是全面建成小康社会、实现第一个百年奋斗目标的决胜阶段，也是我市转型发展、走在前列的攻坚时期。我们要深入贯彻落实党的十八大和十八届三中、四中、五中、六中全会以及习近平总书记系列重要讲话重要批示精神，围绕中央“五位一体”总体布局和“四个全面”战略布局，按照市第十二次党代会对今后五年工作的部署要求，坚定不移践行创新、协调、绿色、开放、共享发展理念，坚定不移深化供给侧结构性改革，坚定不移落实“一个定位、三个着力”总体思路，坚定不移推进“四位一体、组群统筹、全域融合”，奋力谱写现代化组群式大城市建设新篇章。

根据市第十二次党代会确定的发展目标，结合市“十三五”规划提出的目标任务，到2021年，全市地区生产总值达到6300亿元以上，一般公共预算收入突破500亿元；固定资产投资、社会消费品零售总额年均分别增长11%、12%；城镇居民和农村居民人均可支配收入年均分别增长7％和7.8％，在全面建成小康社会进程中走在前列。工作落实上，全市各级政府将重点围绕六大任务集中用力、加速突破：

——聚力打造创新驱动力强、产业层次高、集约集群优势突出的工业强市。把创新驱动作为经济发展的主引擎，以山东半岛国家自主创新示范区（淄博园）建设为统领，全力创建国家创新型城市。深度实施工业精准转调，围绕改造提升七大传统产业、培育壮大五大战新产业、推动二三产业融合发展，集中实施新一轮技术改造三年计划和战新产业新一轮倍增计划，着力培育一批行业骨干企业和创新型高成长企业，产业智能化、绿色化、品牌化达到全省先进水平。抓好市级统筹的六大园区和九个省级经济开发区建设，打造10个左右主营业务收入200-500亿元的专业化产业基地，加快形成主导产业突出、龙头企业带动、产业链条完整、集约集群发展格局。到2021年，全市规模以上工业主营业务收入突破1.5万亿元，高新技术产业占比达到37%，服务业占比达到50%以上，园区经济占工业经济的比重达到80%，主营业务收入过百亿元的企业达到20家以上，全社会研发投入占比达到2.75%，基本建立起以高新技术产业为主导、先进制造业和现代服务业为支撑的现代产业体系。

——聚力打造特色鲜明、品牌引领、文旅融合的文化名城。深入挖掘阐发齐文化丰富内涵，统筹整合鲁商文化、聊斋文化、孝文化、陶琉文化、渔洋文化、红色文化、黄河文化等特色文化资源，重点培育姜太公、管仲、稷下学宫、聊斋等文化品牌，齐文化传承创新示范区建设取得显著成效。扎实推进文化旅游融合发展“十大工程”，积极创建5A级景区，推动“齐国故都与齐王陵”申报世界文化遗产，到2021年，全市旅游消费总额达到880亿元，文化产业增加值占比达到5%以上。健全提升覆盖城乡的公共文化服务体系。继续保持全国文明城市称号。

——聚力打造水清气爽、宜业宜居、人与自然和谐的生态淄博。以建设国家生态文明先行示范区为载体，严格落实《淄博市生态红线保护规划》，坚定不移推进大气、水和土壤污染防治，高标准实施一批环境治理、生态修复和生态绿化工程，加快构建主城区“八河联通、六水共用、清水润城”生态水系，以及“南蓄北引、三河相通、两库相联、客水补源”全域一体生态水系，全面完成国家和省下达的节能减排目标任务。到2021年，全市万元GDP能耗和环境空气质量综合指数均达到全省平均水平，大气四项主要污染物浓度均累计下降30%以上，优良天数和“蓝繁”天数占比达到80%；境内流域水系全部恢复生态功能，70%以上达到Ⅲ类水体；森林覆盖率和建成区绿化覆盖率分别达到38%和47.5%以上，让天蓝地绿、水清气爽成为淄博城市新形象、市民生活“新常态”。

——聚力打造“四位一体、组群统筹、全域融合”的现代化组群式大城市。全力推进淄博新区、高铁新城、淄博经济开发区、文昌湖旅游度假区建设发展，带动形成张店（含淄博高新区）、周村（含淄博经开区）、桓台、文昌湖旅游度假区“四位一体”发展格局，凸显主城区引领带动作用；推进淄川、博山、临淄与“四位一体”统筹发展，形成城市发展紧密圈层；推进高青、沂源与整个组群式城市的融合发展，形成全域“一盘棋”格局。组织实施城市轻轨以及张沂高速、济淄潍高速、小清河复航淄博段等一批关系全局和长远的重大项目，构建起市域“目”字形高速路网、主城区“井”字形快速通道和“十”字形轨道交通网络。扎实推进海绵城市、坚韧城市和智慧城市建设，显著提升城市功能和内涵品质，努力实现传统城市向现代城市转型跨越。到2021年，全市常住人口和户籍人口城镇化率分别达到73%和65%以上，85％以上的村庄达到美丽乡村建设标准。

——聚力打造公共服务更加均衡、人民生活更加殷实的幸福城市。牢固树立以人民为中心的发展思想，深入推进国家扶贫改革试验区建设，下大气力解决教育、医疗、社保、养老等重点领域的民生问题，基本完成城市棚户区、老旧住宅小区、背街小巷改造整治和农贸市场建设，全面完成农村“五化”工程，促进社会事业高位均衡发展，让科学发展成果更加充分地体现在群众幸福感和满意度提升上。到2020年所有贫困人口与全市人民同步实现全面小康。法定人群实现应保尽保；高水平普及15年基础教育；镇办、村居医疗卫生服务机构标准化建设达到95%以上；人均拥有公共文化体育设施面积达到2.2平方米。

——聚力打造改革创新、开放融合的活力城市。把中央和省深化改革决策部署与我市转型发展实际相结合，着力在产业升级、财税金融、生态环保、社会治理、营商环境、改善民生等关键领域推出一批利当前惠长远的改革举措，巩固提升“三最”城市建设成果，法治淄博、平安淄博、诚信淄博建设深入推进，全社会创业创新活力充分释放，群众获得感明显增强。扩大更高水平对外开放，积极融入“一带一路”及全省“两区一圈一带”等区域发展战略，组织实施“大院、大所、大校、大企”招引工程，下大气力引进一批支撑转型升级的科研机构、优质项目、高端人才和创新团队。加大外贸优进优出力度。到2021年，全市进出口总额突破1000亿元，市场主体数量突破50万户，努力构建具有区域竞争优势的开放发展新格局。

三、2017年经济社会发展目标任务和工作重点

2017年是实施“十三五”规划的重要一年，也是供给侧结构性改革的深化之年，全市经济社会发展预期目标建议为：地区生产总值增长7.5%左右，一般公共预算收入增长8.5%左右，固定资产投资增长12%左右，社会消费品零售总额增长11%左右，外贸进出口、实际到账外资保持稳定，城镇居民和农村居民人均可支配收入分别增长8%左右和8.5%左右，城镇登记失业率控制在4%以内，人口自然增长率8.7‰左右，全面完成省下达的节能减排约束性目标。围绕实现上述目标，要牢牢把握稳中求进工作总基调，按照“一个定位、三个着力”总体思路和实现“十个新突破”任务要求，扎实做好七个方面的重点工作：

（一）突出供给侧结构性改革，加快构建现代产业体系

一是扎实推进工业精准转调。进一步细化落实工业精准转调“1+N”实施方案，着力打造26条产业创新链。突出工艺改进和装备升级，集中抓好计划总投资610亿元的280个重点技改项目。加快建设一批智能工厂和智慧车间，努力在自动化、信息化、智能化改造上实现突破。深入推进“互联网+”专项行动，推动传统制造企业从单纯生产加工向个性化研发、产品维护监测、整体方案设计等服务环节延伸。切实抓好新材料、电子信息、生物医药、新能源汽车、动力电源、光伏产业、制药装备等领域一批重大项目，高新技术产业占比提高1个百分点以上。坚定不移淘汰低端落后产能，认真做好去产能过程中职工安置、培训、分流再就业工作。努力创建全国老工业城市产业转型升级示范区。

二是加速发展现代服务业。大力发展生产性服务业，突出抓好远成物流中心、传化金泰公路港、齐鲁股权交易中心、陶瓷产业总部基地和高新区MEMS产业孵化器等一批服务业聚集区，培育壮大卓创资讯、乐物网等骨干电商平台，为产业创新发展提供全要素服务。积极适应和引领社会消费需求，推动教育培训、文化娱乐、体育休闲、健康养老、家政服务等生活性服务业实现更大发展。集中抓好年度投资224亿元的148个市级服务业重点项目，全市服务业增加值占比提高1.5个百分点以上。

三是深化农业供给侧结构性改革。在稳定粮食生产的基础上，把增加绿色优质农产品供给放在突出位置，以农产品质量安全市、农业综合执法规范市、食品安全市“三市”同建为抓手，实施“农业转型升级十大行动”，着力发展优质专业生产基地，扩大“三品一标”农产品规模。积极推进“互联网+现代农业”，加快发展智慧农业。细化落实承包土地“三权分置”办法，鼓励发展多种形式的适度规模经营，推动农产品生产加工与都市农业、乡村旅游融合发展，带动农业增效和农民增收。

（二）突出打造优质载体，加快培育转型升级新动力

一是深入推进创新驱动。以山东半岛国家自主创新示范区（淄博园）建设为突破口，组织实施高新技术企业培育计划、国家“创新百强”工程和制造业单项冠军培育提升工程，支持企业建设工程技术研究中心、重点实验室、院士工作站、博士后科研工作站等创新平台，切实增强产业创新联盟协同创新能力，全社会研发投入占比达到2.3%，新增省级以上创新平台20家以上。落实鼓励“零成本创业”等政策措施，推动校城融合发展，建设完善齐鲁创业创新谷、创业孵化中心、淄博创业大学等优质载体，支持闲置商业地产向发展“双创”等新业态转化，充分激发全社会创新创业活力。

二是切实强化重大项目带动。集中抓好年度计划投资1026亿元的330个市重大项目，完善落实“三个跟着走”的推进机制，确保项目年度开工率和投资完成率达到100%。创新政银企合作机制，健全政策性融资担保体系，着力提高企业抵质押能力，强化对重大项目、重点工程的融资信贷支持。

三是加快产业布局调整。以市和区县15个重点经济园区为主平台，严格落实园区建设和产业发展规划，抓紧抓实龙头企业培育、重大项目招引、产业链条拉伸、配套功能提升、体制机制创新等关键环节，促进产业集约集聚发展。加快化工产业布局调整，基本完成建陶、耐材产业布局调整和压减产能任务，集中打造氟硅新材料、新能源汽车、医药健康、电子信息、高效电机泵业、高端建陶、高性能耐材等一批优势产业基地。结合永久基本农田划定和推进“多规合一”，进一步抓好经济园区和重大项目用地保障，盘活低效建设用地，拓展产业发展空间。

四是大力提升企业素质。突出“双50强”及各行业具备领军素质的重点企业，着力培育一批技术指标“领跑者”和“单项冠军”，形成一批占据产业高端、掌握核心技术、竞争优势突出的企业集群。推动建立现代企业制度，年内完成300家以上规模企业公司制改制，在齐鲁股交中心挂牌企业达到500家，主板和新三板上市挂牌企业分别达到28家和55家。在重点行业全面推行企业管理对标，大力弘扬精益求精的工匠精神，深入实施品牌战略，开展质量提升行动，加快培育一批国内外知名品牌。扎实推进企业家和高技能人才两个“十年培训计划”，深化职业教育产教融合、校企合作，为企业做大做强提供有力支撑。

（三）突出优势资源整合提升，加快文化旅游融合发展

一是全力打造齐文化传承创新示范区。落实示范区建设发展规划，高水平办好齐文化研究院、齐文化节、稷下论坛和“起源地杯”国际足球邀请赛。加快齐故城考古遗址公园建设，规划建设稷下学宫模拟展示馆，联合省新闻出版广电局创作拍摄大型文献纪录片《稷下学宫》，努力将齐文化打造成为标志性城市品牌。加快发展文化创意、艺术博览、动漫游戏、数字传媒等新型文化业态，办好陶博会和新材料技术论坛等品牌展会，文化产业增加值占比达到4.3%。

二是集中打造旅游目的地城市。以创建国家全域旅游示范区为目标，突出抓好齐长城生态旅游带、陶琉古镇、鲁商示范园等29个重点项目，优化提升10处乡村旅游连片发展区，建立完善贯通全产业链的配套服务体系，着力构建东部齐文化核心区、南部山林生态、中西部特色文化、中北部水系慢城四条精品线路，打造国内知名旅游目的地城市。

三是加大文化惠民力度。突出抓好市文化中心和一批区县重点文化项目，新建7个、扩建1个区县国家综合档案馆，提升完善30个镇办和1200个村居（社区）文化基础设施，构建“15-20分钟”城乡公共文化服务圈。高度重视文物保护工作，切实抓好“乡村记忆”工程，更好地延展城市文脉。

（四）突出深度精准治理，加快改善区域生态环境

一是持续深化大气和水污染防治。坚持“九个一律”，在深化电力、化工、钢铁、焦化等行业治理的同时，集中力量对建陶、耐火材料、日用玻璃、铸造、板簧、氧化铝球、水泥粉磨、碳素等重点行业，以及主城区东部、南部、东北、西南四个重点区域进行大气环境综合整治。加大空气异味治理力度，确保494家重点企业全部实现挥发性有机物达标排放。深入实施绿动力提升工程，2036个已竣工项目全部完成第三方验收并达标运行，续建和新开工的1381个项目上半年全部完工。年内全市空气质量优良天数和“蓝繁”天数分别达到55%和65%以上，改善率继续保持全省前列。对十大涉水重点行业实行企业清洁生产审核，所有直排企业按照COD40mg/L、氨氮2mg/L实施提标改造。抓好工业集聚区污水集中处理、城镇污水处理厂提标改造等基础设施项目，年内市域河流全部消除劣Ⅴ类水体。

二是进一步加强生态修复治理。全面推行“河长制”，加快推进孝妇河、范阳河、东猪龙河等重点流域综合整治，抓好引水入萌、主城区南部水系联通等重点工程，构建“清水润城”生态水系。组织实施“1256”生态修复绿化工程，建成孝妇河湿地公园、玉龙湖湿地公园、文昌湖环湖公园等一批生态景观。开工建设化工集中区危废综合处置中心和填埋场，切实加强土壤修复、重金属污染治理和农业面源污染防治，以最严的措施抓好饮用水水源地保护，确保生态环境安全。

三是健全完善生态建设长效机制。深入推进生态红线划定工作，严格实行环境总量控制、环境影响评价等制度，实现全市镇办空气质量自动监测和市控以上重点断面、涉水重点企业水质自动监测两个“全覆盖”。坚持完善联动执法、异地执法、环保夜查等常态化监管机制，加大“刑责治污”力度，依法严惩环境违法行为。

（五）突出组群城市特色，加快统筹融合发展

一是进一步做实城市总体布局。牢牢把握建设现代化组群式大城市发展定位，编制完善城市发展战略规划、新一轮城市总体规划、重点区域控制性详细规划和各类专项规划，集中抓好淄博新区文化中心、商务金融中心、教育医疗等重大功能区块建设，用两年时间建成高铁新城基础设施和服务体系，增强主城区辐射带动能力。强化主城区与各区域在功能定位、产业布局、基础设施、公共服务、生态体系等方面的统筹协调，突出抓好新生小城市、重点示范镇小城市试点建设和特色小镇培育，加快构建“四位一体、组群统筹、全域融合”发展格局。

二是全面提升综合承载功能。完成城市轻轨前期工作，扎实推进济青高铁、济青高速、滨莱高速和长深高速淄博段建设改造，加快启动张沂高速、张博铁路和淄东铁路淄博段电气化改造及小清河复航淄博段工程，集中实施G309淄博段等10个普通国省道建设改造项目，抓好人民路改造以及城市立交桥等30项重点路网工程，着力完善现代化综合交通体系。建成投用第二环保热电厂，统筹实施地下综合管廊建设、城市供水系统改造，以及总投资40亿元的特超高压等电网建设工程；建设主城区公共自行车系统；全面完成公交集约化改革。把“三改一建”作为推动老城区有机更新的重要抓手，集中抓好144个棚户区、107个老旧住宅小区和16处农贸市场改造建设，全面完成背街小巷整治任务，实现小区物业管理全覆盖。深入开展城市违建、市容市貌和城市秩序专项治理，实施新型智慧城市建设工程，不断提高精细化、智能化、网格化管理水平。

三是积极推进“人的城镇化”。深化户籍制度改革，加快镇改办、村改居工作，着力推进以教育、医疗等为重点的基本公共服务均等化，吸引更多进城务工人员落户城市。加快淄博经开区、文昌湖区、高铁新城、区县经济开发区等重点片区旧村改造，实施重化产业园区村庄搬迁，将符合条件的新型农村社区纳入城镇管理，全市常住人口城镇化率提高1.5个百分点。深入推进美丽乡村标准化建设和农村“五化”工程，基本完成农村旱厕改造任务，让城乡群众共享新型城镇化发展成果。

（六）突出增强区域活力，加快推进改革开放

一是全面深化重点领域改革。着力深化投融资体制改革，运作好城市资产运营、金融控股、文化旅游等投融资平台，推动9支市级政府引导基金全部实现市场化、专业化运作。深化财税金融体制改革，增强政府预算统筹调控能力，兑现落实“工业强市30条”及各级降低企业成本的政策措施。抓好新型农村合作金融试点，与齐鲁股交中心共同建设多层次资本市场联合培育基地。继续推进事业单位改革。进一步深化农村产权制度、市属国有企业、商事制度、价格机制、统计体制等领域改革。

二是大力发展开放型经济。聚焦世界500强和行业领军企业，集中招引一批带动力强的大项目、好项目，年内全市实际到位外来投资达到900亿元。坚持引资引智并重，抓住用好国家疏解非首都功能和深化军民融合发展的重大机遇，扎实推进“四大”招引工程，全力争取中国钢铁研究院、中科院过程工程研究所、北京有色金属研究总院等国家级科研机构进驻淄博，布局建设国内一流专业化产业基地。推动淄博保税物流中心申建综合保税区。年内全市新增外贸进出口实绩企业300家以上，进出口总额突破100亿美元。

三是切实打造一流营商环境。深化“放管服”改革，继续精简规范行政审批事项，全面完成“红顶中介”脱钩任务，推动网上审批和公共服务向镇办延伸，始终保持动态“三最”。健全“双随机、一公开”监管机制，既要切实加强对市场主体的事中事后监管，又要坚决查处纠正各类不作为、乱作为。加快市电子政务和信息中心建设，开工建设市云计算中心，完善提升全市统一的公共资源交易平台，建成覆盖主要经济社会管理部门的“信用淄博”公共信息平台。

（七）突出补齐发展“短板”，加快民生和社会建设

一是统筹做好扶贫就业和社保工作。加快建立长效脱贫机制，将市定扶贫线和低保线统一提高至人均每年4200元，确保市定标准贫困人口稳定实现“两不愁、三保障”。坚持以创业带动就业，应届高校毕业生总体就业率稳定在85%以上，城镇新增就业7万人以上。扎实推进“全民参保登记计划”，切实落实各项社保政策。进一步完善困难群众兜底保障和社会救助体系，加快推动社会保障由制度全覆盖到人群全覆盖。

二是提高教育、卫生、养老等社会事业发展水平。积极支持山东理工大学创建中国一流大学，扎实推动齐鲁医药学院新校区、淄博新区普通高中、淄博十七中等重大项目建设，完成30所中小学和52所幼儿园新改扩建，切实解决城镇中小学“大班额”问题，提前一年完成“全面改薄”任务。深化城市公立医院综合改革，抓好市中心医院西院区、市妇幼保健院新院区等总投资30.5亿元的12个重点项目，启动社区卫生服务中心标准化建设，努力创建全国基层中医药工作先进市，加快建设健康淄博。推进社区养老示范工程，年内城市社区日间照料中心达到200家、农村养老服务设施新增100家。

三是加强和创新社会治理。强化底线思维和红线意识，完善“党政同责、一岗双责、齐抓共管、失职追责”安全生产责任体系，持续开展综合整治和专项治理，严查彻治各类安全隐患；坚定不移推进“刑责治安”，严格落实“一票否决”制度，对违法生产企业坚决关停整治，对责任人员坚决依法依纪追责。大力加强食品药品监管，努力创建省级食品安全城市。强化金融领域和实体经济运行监测，全力推进重点担保圈综合治理，依法打击恶意逃废银行债务、非法集资、新型电信网络诈骗等违法犯罪行为，坚决守住不发生区域性金融风险的底线。加强普法依法治理，支持人民法院解决“执行难”问题。健全信访维稳和矛盾纠纷多元化解机制，扎实推进“六型警务”建设，完善立体化社会治安防控体系，确保社会大局和谐稳定。

四、切实加强法治政府和服务型政府建设

一是更加自觉地坚持依法行政。注重运用法治思维谋划发展、制定政策、推动工作，完善重大行政决策专家咨询、法律顾问制度和目录管理制度，增强行政决策的规范化和透明度。深化行政执法体制改革，持续推动执法重心和执法力量下移，做到严格规范公正文明执法。认真落实市人大及其常委会决议决定，自觉接受人大法律监督、政协民主监督和社会舆论监督，积极听取民主党派、人民团体和社会各界人士的意见建议，扎实推进覆盖政府工作全过程的政务公开和重点领域信息公开，确保权力在阳光下运行。

二是更加深入地推进从严治政。切实增强政治意识、大局意识、核心意识、看齐意识，严守纪律规矩，坚定立场方向，确保政令畅通、令行禁止。完善落实政府权力清单、责任清单和负面清单，加强对公共资金、国有资产、国有资源的审计监督，坚决做到有权必有责、用权受监督、失职要问责、违法要追究。抓严抓实党风廉政建设，扎紧制度笼子，持续加大反腐败力度，集中整治项目招投标、土地招拍挂和政府采购中的违法违规问题，严肃查处顶风违纪行为，坚决将从严治党、从严治政要求落到实处。

三是更加有力地强化责任担当。大力践行“六个干”工作要求，敢于直面矛盾，始终保持奋发有为、干事创业的精气神。完善正向激励和容错机制，旗帜鲜明地为担当者担当、为负责者负责。切实树立问题导向，紧紧抓住转型发展的重点领域和关键环节，以目标倒逼责任、以时间倒逼进度、以督查倒逼落实，努力推动各项事业争先进位、走在前列。大力加强作风建设，建立健全密切联系群众制度，及时回应社会和群众关切。坚决兑现对市场主体和人民群众的各项承诺，言必信、行必果，不断提高政府执行力和公信力。

各位代表！美好蓝图催人奋进，历史使命艰巨光荣。让我们更加紧密地团结在以习近平同志为核心的党中央周围，在中共淄博市委的坚强领导下，撸起袖子加油干，为全面建成小康社会、建设现代化组群式大城市而努力奋斗！
